# Adaptive Frequency‐Optimized Wavelet Networks for Early Detection of Subjective Cognitive Decline via Resting‐State fMRI

**DOI:** 10.1002/brb3.71039

**Published:** 2025-12-07

**Authors:** Xiereniguli Anayiti, Yupan Ding, Weikai Li, Mingyu Tan, Peiying Chen, Zhongfeng Xie, Mengling Tao, Yongsheng Xiang, Yingying Liu, Xiaowen Xu, Peijun Wang

**Affiliations:** ^1^ Department of Medical Imaging Tongji Hospital School of Medicine Tongji University Shanghai China; ^2^ Institute of Medical Imaging Artificial Intelligence Tongji University School of Medicine Shanghai China; ^3^ School of Mathematics and Statistics Chongqing Jiaotong University Chongqing China

**Keywords:** Frequency Self‐Adaptive Wavelet Transform, functional connectivity network, subjective cognitive decline

## Abstract

**Background:**

Early detection of subjective cognitive decline (SCD), a preclinical stage of Alzheimer's disease (AD), remains a clinical challenge due to its subtle manifestations. This study aims to address these challenges by introducing a novel approach to enhance the detection and analysis of SCD.

**Methods:**

A Frequency Self‐Adaptive Wavelet Transform (FSAWT) model was developed and optimized for functional brain network (FBN) construction using resting‐state functional MRI (rs‐fMRI) data. The model dynamically selected “golden frequencies” to improve the accuracy and interpretability of brain connectivity patterns. FBNs from 240 participants (106 SCD, 134 controls) were analyzed and compared using traditional methods, pearson correlation (PC) and sparse representation (SR). Receiver operating characteristic‐area under the curve (ROC‐AUC) analysis validated the classification results.

**Results:**

Our findings demonstrate that individuals with SCD exhibit distinct functional connectivity alterations, including reversed parahippocampal gyrus‐superior parietal gyrus connectivity—suggesting early DMN disintegration, weakened temporoparietal pathways linked to memory deficits, and enhanced fusiform gyrus‐orbitofrontal connectivity. The frequency‐optimized SRWT method achieved superior diagnostic performance (83.71% accuracy, AUC = 0.84) with 82.11% sensitivity and 85.71% specificity, significantly outperforming traditional approaches (61.93% accuracy for PC), highlighting its potential for early SCD detection through these network‐based biomarkers.

**Conclusions:**

The FSAWT model offers a robust framework for early SCD detection by integrating frequency‐specific and cross‐frequency dynamics. While these findings highlight potential contributions to precision diagnostics and personalized interventions for neurodegenerative disorders, such applications remain to be established in future studies. Future applications may also explore multimodal neuroimaging and broader cognitive impairments.

## Graphical abstract

Our Frequency Self‐Adaptive Wavelet Transform (FSAWT) model improves early detection of subjective cognitive decline (SCD) from rs‐fMRI. It identifies distinct network hubs and altered low‐frequency connectivity within the DMN and sensorimotor networks.

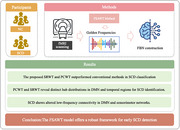

**Abbreviations**
AAL: automated anatomical labeling atlas
AD: Alzheimer's disease
ADL: activities of daily living
AMYG: amygdala
BOLD: blood oxygen level‐dependent
CAL: calcarine cortex
CAU: caudate
CNM: coordinate network mapping
CTCN: cingulo‐opercular task control network
CUN: cuneus
DMN: default mode network
FBN: functional brain network
FFG: fusiform gyrus
FSAWT: Frequency Self‐Adaptive Wavelet Transform
FTCN: frontoparietal task control network
HAMA: Hamilton Anxiety Rating Scale
HAMD: Hamilton Depression Rating Scale
HIP: hippocampus
IFGoperc: inferior frontal gyrus, opercular part
INS: insula
IOG: inferior occipital gyrus
IPL: inferior parietal lobule
ITG: inferior temporal gyrus
LING: lingual gyrus
LOO‐CV: leave‐one‐out cross‐validation
MCI: mild cognitive impairment
MMSE: Mini‐Mental State Examination
MOG: middle occipital gyrus
MTG: middle temporal gyrus
NCs: normal controls
OLF: olfactory cortex
ORBmid: middle orbital gyrus
ORBsup: superior orbital gyrus
ORBsupmed: superior medial orbital gyrus
PC: Pearson correlation
PCL: paracentral lobule
PCUN: precuneus
PCWT: Pearson Correlation Wavelet Transform
PHG: parahippocampal gyrus
PoCG: postcentral gyrus
PUT: Putamen
REC: Rectus Gyrus
ROC‐AUC: receiver operating characteristic‐area under the curve
ROL: Rolandic operculum
rs‐fMRI: resting‐state functional MRI
SCD: subjective cognitive decline
SCD‐Q: Subjective Cognitive Decline Questionnaire
SFGmed: superior medial frontal gyrus
SMN: somatosensory network
SN: salience network
SPG: superior parietal gyrus
SR: sparse representation
SRWT: Sparse Representation Wavelet Transform
STG: superior temporal gyrus
VN: visual network

## 1 Introduction

Alzheimer's disease (AD) is a progressive neurodegenerative disorder characterized by cognitive decline and memory impairment. Subjective cognitive decline (SCD), defined as a self‐perceived worsening of cognitive function despite normal performance on standardized tests, is increasingly recognized as the earliest symptomatic stage of AD. Frank Jessen and colleagues proposed a framework for SCD, recognizing it as a key preclinical stage of AD. It precedes mild cognitive impairment (MCI) and overt dementia, providing a potential window for timely interventions that may slow or prevent disease progression (Jessen et al. 2014; van Harten et al. 2018). Studies show that individuals with SCD, especially those with amyloid pathology, are at increased risk of progressing to MCI and AD. It is estimated that 14% of SCD individuals will develop dementia, and 27% will progress to MCI, highlighting SCD's importance in early diagnosis and intervention (Jessen et al. 2020, 2014). Recent evidence has further validated SCD—particularly in individuals with underlying amyloid pathology—as a distinct molecular and clinical stage (Stage 2) within the AD continuum, supported by elevated plasma biomarkers such as phosphorylated tau (p‐tau181), which predict both cognitive decline and progression to MCI (Mengel et al. 2025). In addition, plasma p‐tau217 has shown high accuracy in detecting amyloid pathology even in individuals with SCD (Gonzalez‐Ortiz et al. 2023). Multicenter data from the DELCODE cohort further demonstrated that amyloid‐positive SCD cases present greater hippocampal atrophy, subtle cognitive and functional decline, and behavioral symptoms compared to controls, supporting their classification as Stage 2 of the AD continuum (Jessen et al. 2023). Beyond fluid biomarkers, neurophysiological and imaging studies also reveal early SCD alterations. Resting‐state EEG shows increased theta power, though other bands remain inconsistent (Perez et al. 2024). Structural magnetic resonance imaging (MRI) demonstrates subtle gray matter and cortical thinning in frontal, parietal, and medial temporal regions, resembling MCI and AD (Rivas‐Fernández et al. 2023). A recent review further highlighted advances in risk factors and biomarkers, while stressing the need for more sensitive tools to detect early brain changes (Munro et al. 2023).

Resting‐state functional MRI (rs‐fMRI) has emerged as a valuable technique for examining intrinsic brain activity. By capturing spontaneous fluctuations in the blood oxygen level‐dependent (BOLD) signal, rs‐fMRI allows for the construction of functional brain networks (FBNs), which reflect large‐scale patterns of functional integration and segregation. Altered FBN organization has been reported across the AD continuum, including in SCD individuals, particularly within the default mode network (DMN), hippocampus (HIP), and other memory‐related regions (Liu et al. 2024; Wang et al. 2022). Recent evidence using coordinate network mapping (CNM) reveals that despite heterogeneous neuroimaging findings in individuals with SCD, these abnormalities consistently converge on specific large‐scale brain networks—primarily the DMN and the somatosensory network (SMN). Furthermore, transcriptomic and neurotransmitter decoding linked these networks to molecular pathways associated with AD and neuropsychiatric dysfunction, offering cross‐modal evidence for their role in early pathological processes (Lan et al. 2025). Moreover, studies have used brain network analysis to successfully differentiate SCD from cognitively normal controls (NCs) (X. Xu et al. 2022). While previous studies have identified brain network alterations in SCD, these studies have often relied on traditional connectivity methods. Our study is unique in applying the Frequency Self‐Adaptive Wavelet Transform (FSAWT) to capture dynamic, frequency‐specific connectivity, offering greater sensitivity in distinguishing individuals with SCD from cognitively normal adults.

Traditional FBN construction techniques, such as Pearson correlation (PC) and sparse representation (SR), have limitations in capturing the dynamic and frequency‐specific characteristics of brain connectivity. These methods typically assume static connectivity and often rely on fixed frequency bands, potentially overlooking critical cross‐frequency interactions and region‐specific spectral features (Ding et al. 2023; Zhou et al. 2010). To address these limitations, we propose a novel FSAWT model that dynamically identifies optimal frequency bands—termed “golden frequencies”—for each brain region or connection. By enhancing the sensitivity of FBNs to subtle alterations in SCD, FSAWT may facilitate earlier and more accurate detection of preclinical AD‐related changes, highlighting its potential for detecting early cognitive decline, as demonstrated in previous studies (Ding et al. 2023).

This study aims to identify FBN alterations associated with SCD and to distinguish individuals with SCD from cognitively normal adults. By exploring key brain regions and connectivity patterns linked to SCD, our findings may contribute to earlier identification and provide insights for targeted interventions in the preclinical stages of neurodegeneration.

## 2 Materials and Method

### 2.1 Study Participants

This study used data collected from Tongji Hospital, affiliated to Tongji University in Shanghai, between January 2020 and December 2023. A total of 240 participants were included in the final analysis, comprising 134 NCs and 106 individuals with SCD. All participants were evaluated by experienced clinicians and underwent a comprehensive clinical and neuropsychological assessment.

Participants in the SCD group met criteria consistent with the international SCD research framework (Jessen et al. 2014). Specifically, SCD was defined by the presence of self‐reported cognitive complaints, particularly involving memory decline, which were assessed using both a structured clinical interview—including the question “Do you feel your memory has declined compared to a few years ago?”—and the Subjective Cognitive Decline Questionnaire (SCD‐Q), with a total score ≥ 7 indicating significant complaint (Rami et al. 2014). Importantly, all participants exhibited normal objective cognitive performance, with Mini‐Mental State Examination (MMSE) scores ≥ 26 (adjusted for age and education). They did not meet the criteria for MCI or dementia based on structured clinical interviews and neuropsychological testing. To minimize the influence of psychiatric comorbidities, anxiety and depressive symptoms were evaluated using the Hamilton Anxiety Rating Scale (HAMA) and Hamilton Depression Rating Scale (HAMD). Participants scoring ≥ 7 on the HAMA and HAMD were excluded (Canadian Agency for Drugs and Technologies in Health 2016).

The NC group had no subjective cognitive complaints, scored within the normal range on cognitive assessments (MMSE ≥ 26), exhibited no functional impairments, and had no history of neurological or psychiatric disorders.

Common exclusion criteria for both groups included: (1) current or past major psychiatric disorders; (2) neurological conditions such as stroke, epilepsy, or traumatic brain injury; (3) substance abuse or dependence; and (4) inability to complete the MRI protocol or any contraindications for MRI scanning.

The study was approved by the Ethics Committee of Tongji Hospital, affiliated to Tongji University. All participants provided written informed consent prior to enrollment, in accordance with the Declaration of Helsinki.

### 2.2 MRI Data Acquisition and Preprocessing

The rs‐fMRI data were acquired using a 3.0T Siemens Magnetom Verio MRI scanner with a 32‐channel head coil. Participants were instructed to keep their eyes closed, stay awake, and minimize movement. The scanning parameters included a TR of 2000 ms, TE of 30 ms, a flip angle of 90°, a matrix size of 64 × 64, and an FOV of 224 × 224 mm^2^. Preprocessing was performed using SPM and DPARSFA (v2.2). The first 10 images were discarded to ensure signal stability. Steps included slice timing correction, motion correction, spatial normalization to a standard template, and smoothing to improve signal‐to‐noise ratio. High‐pass filtering removed low‐frequency noise, while covariate regression and detrending controlled for confounds and linear drift. Signal intensity was then standardized across the dataset.

### 2.3 Methods in Brain Network Analysis

To explore brain connectivity alterations in individuals with SCD, we constructed subject‐specific FBNs based on rs‐fMRI data. Each participant's brain was parcellated into regions of interest (ROIs), and functional associations between ROIs were estimated using correlation‐based and model‐driven approaches.

PC was first applied to capture pairwise temporal synchronization between ROIs. However, to reduce the influence of spurious connections inherent in fully connected networks, thresholding and regularization strategies were employed. To further refine connectivity estimates, partial correlation analysis was adopted, enabling the assessment of direct functional relationships while accounting for confounding signals from other brain regions. Sparse regression techniques, including LASSO, were used to address the high‐dimensional nature of fMRI data and limited time series length.

**FIGURE 1:**
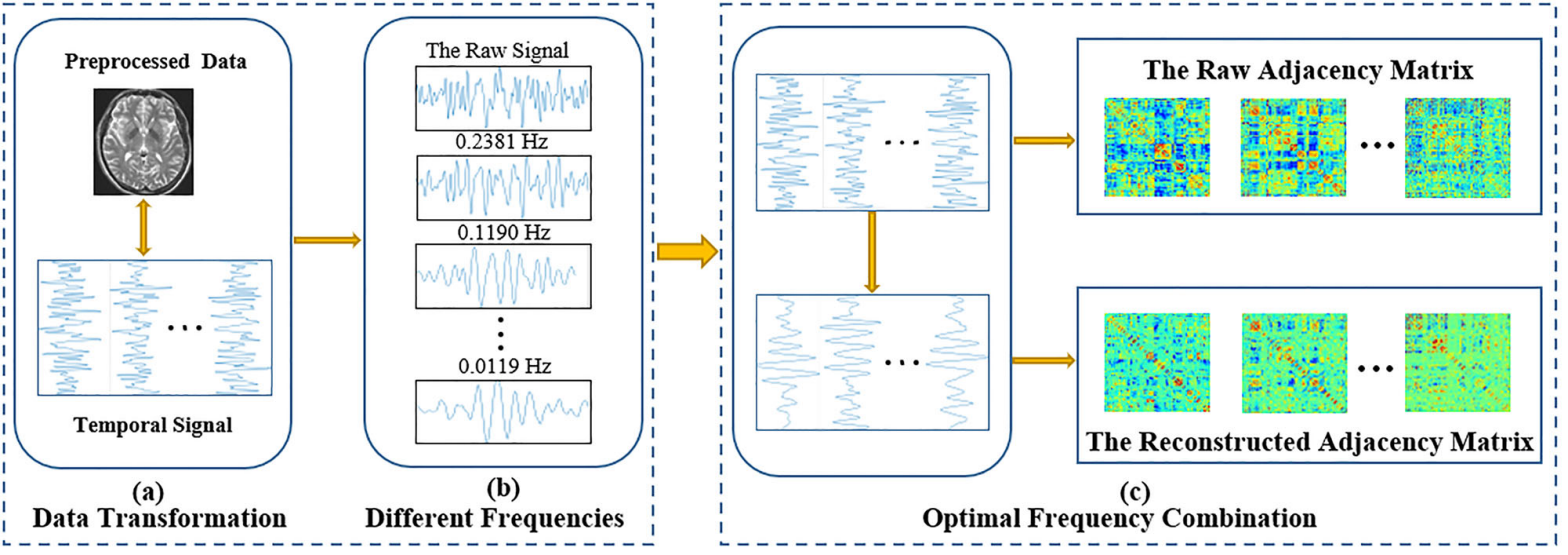
Flowchart of FSAWT method. (a) A preprocessing operation is performed on data to convert it into time series signals. (b) Each time series signal is decomposed into different frequency signals by the wavelet transform. (c) The optimal frequency combination is then selected based on the maximum Pearson coefficient, following which the partitioned clear adjacency matrix is constructed, and the original time series signal is reconstructed.

In addition, we incorporated time–frequency decomposition via Continuous Wavelet Transform (CWT) to capture transient and scale‐specific dynamics in the BOLD signal, which may reflect subtle functional disruptions associated with SCD. In all group‐level connectivity analyses, age, sex, and years of education were included as covariates to control for potential demographic confounds. Figure 1 presents the workflow and fundamental concept of the FSAWT method. A detailed description of network construction procedures, mathematical formulations of network matrices is provided in Supporting Information (1.1) and Figure S1.

### 2.4 Functional Network Optimization via Frequency Self‐Adaptive Wavelet Transform

Traditional FBN construction methods based on fixed frequency bands or sinusoidal decomposition may fail to capture transient, nonstationary features in resting‐state fMRI signals, potentially distorting network topology. To address this, we propose a FSAWT method, which adaptively selects optimal frequency components for each ROI to enhance the reliability and modularity of FBNs.

This method decomposes each ROI's time series into multiple frequency bands using CWT, and constructs optimized adjacency matrices by selecting combinations that maximize interregional correlations. By grouping ROIs and reducing the search space via a scalable partitioning strategy, the method significantly lowers computational complexity while improving matrix clarity. The resulting FBNs show improved modularity and reduced noise compared to conventional PC‐based networks. Each ROI's time series is decomposed into multiple frequency bands using CWT, which allows for the analysis of both low‐ and high‐frequency oscillations. The interregional correlations between ROIs are calculated across these frequency bands. The optimal frequency combination is selected by maximizing the PC between ROI pairs, ensuring that the resulting adjacency matrix reflects the most significant functional connectivity patterns. This process involves evaluating correlations for each frequency band and selecting those that provide the strongest relationships between regions, which enhances the clarity and reliability of the brain network construction.

A full description of the model formulation, optimization algorithm, and experimental workflow—including mathematical derivations, gradient descent procedure, and the proximal operator—is provided in Supporting Information (1.2) and Figure S2.

### 2.5 Experimental Setting

To evaluate the performance of our proposed FSAWT method, we conducted a classification task distinguishing NC from SCD based on fMRI data. All BOLD signals were parcellated into 90 brain regions using the AAL atlas, and feature construction was based on various FBN estimation methods, including traditional PC, SR, and their wavelet‐based extensions (Pearson Correlation Wavelet Transform [PCWT], Sparse Representation Wavelet Transform [SRWT]). To ensure clinical interpretability and robustness, we used a linear classifier with nested leave‐one‐out cross‐validation (LOO‐CV) to avoid overfitting. Functional connectivity features were selected using *t*‐tests at multiple significance levels, and model performance was evaluated in terms of accuracy, sensitivity, specificity, and AUC. Before classification, the effects of age, sex, and years of education were regressed out from all connectivity features to minimize potential confounding. For group‐level analysis, hub nodes were identified from group‐averaged networks by ranking nodal degrees, with the top 5% of regions defined as hubs. More detailed information regarding wavelet scale selection, hyperparameter settings, and frequency decomposition strategies is provided in Supporting Information (1.3).

## 3 Results

### 3.1 Demographic and Clinical Characteristics

The demographic and clinical characteristics of participants in the NC and SCD groups are summarized in Table 1. A total of 240 participants were included, comprising 134 NCs and 106 individuals with SCD. A significantly higher proportion of females was observed in the SCD group compared to the NC group (69.8% vs. 47.0%, *p* < 0.001), indicating a potential gender‐related vulnerability to self‐perceived cognitive decline. In addition, the SCD group was significantly younger than the NC group (*p* < 0.001). No significant differences were found between the two groups in terms of education level (*p* = 0.17), MMSE scores (*p* = 0.63), MoCA scores (*p* = 0.07), or ADL scores (*p* = 0.20).

**TABLE 1:** Demographic and clinical characteristics of NC and SCD participants.

	NC	SCD	*p* value
*N*	134	106	
Gender (male/female, %)	53%/47%	30.2%/69.8%	< 0.001^***^
Age (years)	71 (66–76)	68 (64–72)	< 0.001^***^
Education (years)	12 (9–15)	12 (9–13)	0.17
MMSE score	28 (27–29)	28 (27–29)	0.63
MoCA score	27 (26–27)	26 (26–27)	0.07
ADL score	14 (14–15)	14 (14–14)	0.20

*Note*: Values are presented as median (interquartile range) for continuous variables and percentage for categorical variables. Group differences were assessed using the Mann–Whitney *U* test for continuous variables and chi‐square test for categorical variables.

Abbreviation: ADL, activities of daily living.

^*^ *p* < 0.05, ^**^ *p* < 0.01, and ^***^ *p* < 0.001.

### 3.2 Diagnostic Performance of SCD Classification

As shown in Table 2, the proposed PCWT and SRWT methods significantly outperformed traditional PC and SR approaches in identifying individuals with SCD. SRWT achieved the highest classification accuracy of 83.71%, followed by PCWT at 73.22%. Both methods also demonstrated substantially higher sensitivity (82.11% for SRWT and 82.02% for PCWT), suggesting improved ability to detect true SCD cases and thereby reduce the risk of false negatives. While this highlights the potential relevance of such methods for clinical screening and early intervention, their clinical applicability requires further validation in external datasets and real‐world settings.

**TABLE 2:** Results of different FBN estimation methods on the dataset (where the *p* value is 0.01 because these methods give the best results at this *p* value.).

Method	Accuracy	Sensitivity	Specificity	AUC
PC	61.93%	56.87%	68.57%	0.5789
PCWT	73.22%	82.02%	61.90%	0.7160
SR	64.06%	80.66%	42.86%	0.5371
SRWT	83.71%	82.11%	85.71%	0.8422

**FIGURE 2:**
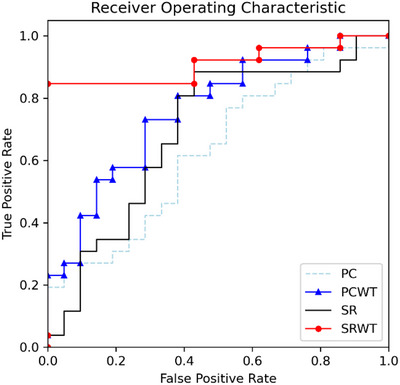
ROC curves for PC, PCWT, SR, and SRWT classification effects.

ROC curve analysis further supported these findings (Figure 2). SRWT and PCWT exhibited greater AUC values compared to PC and SR, indicating enhanced discriminatory power. These results highlight the potential of frequency‐optimized wavelet‐based methods to more effectively differentiate between individuals with and without early cognitive symptoms, which is essential in clinical risk stratification. We further evaluated the robustness of PCWT and SRWT across different parameter settings (see Figures S3 and S4). While SRWT achieved the highest overall accuracy, it also showed greater sensitivity to parameter variation compared to PCWT. These findings suggest that PCWT may offer more stable performance in practical settings, whereas SRWT may require more careful parameter tuning.

### 3.3 Discriminative Connectivity Patterns in FBN Classification

**FIGURE 3:**
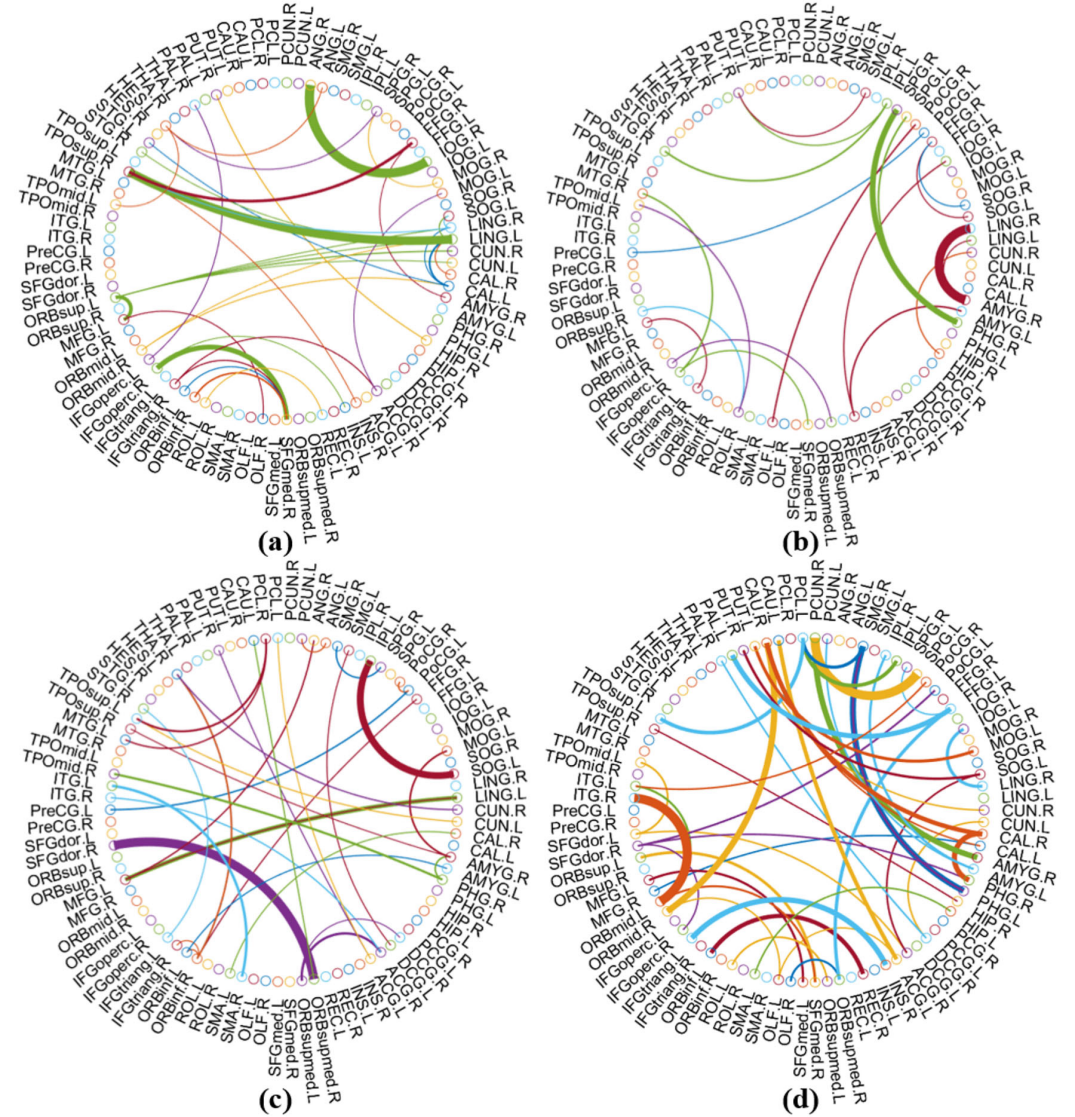
Illustration of the most discriminative connectivity features (based on *t*‐test, *p* < 0.01). The arc color is randomly generated only to distinguish different connections, and the arc thickness represents the discriminative ability of the corresponding connection, which is inversely proportional to the *p* value. (a) Discriminative connections generated by PC. (b) Discriminative connections generated by PCWT (threshold value of 50%). (c) Discriminative connections generated by SR. (d) Discriminative connections generated by SRWT (λ =2^0^).

When building the FBN from fMRI data for classification, it is essential to identify discriminative features, especially with the PC and PCWT methods. This helps highlight key brain regions and compare the two approaches. Using a *t*‐test, we extracted the most distinguishing connections, shown in Figure 3 with Paul Kassebaum's “circular Graph” function, emphasizing their role in the classification model.

Both PCWT and PC focus on connections involving the superior parietal gyrus (SPG), lingual gyrus (LING), and dorsolateral superior frontal gyrus. However, PCWT enhances the importance of the parahippocampal gyrus (PHG) and eliminates extraneous connections like those between the lenticular nucleus and thalamus. This indicates that PCWT more effectively isolates key features, reducing the influence of weakly correlated connections. As shown in Table 3, the top 10 connections identified by PCWT are in the temporal, parietal, and limbic regions, aligning with networks like the DMN, VAN, and hippocampal system.

**TABLE 3:** Top 10 most discriminative connections for PCWT (*t*‐test based on *p* < 0.01).

Connection		NC	SCD	*p* value	Comparison (NC vs. SCD)
Region A	Region B				
AMYG.R	LING.R	0.005 ± 0.036	−0.085 ± 0.232	< 0.001	NC > SCD
PHG.R	SPG.R	−0.039 ± 0.024	0.037 ± 0.029	< 0.001	SCD > NC
IPL.L	STG.R	−0.083 ± 0.036	−0.005 ± 0.033	0.002	SCD > NC
PHG.R	SPG.L	−0.065 ± 0.028	0.003 ± 0.027	0.002	SCD > NC
REC.R	AMYG.L	0.016 ± 0.021	0.088 ± 0.044	0.002	SCD > NC
IFGoperc.R	MTG.R	−0.035 ± 0.051	0.054 ± 0.039	0.002	SCD > NC
OLF.L	PoCG.R	−0.013 ± 0.023	0.056 ± 0.036	0.002	SCD > NC
REC.R	IOG.R	−0.033 ± 0.028	0.031 ± 0.026	0.003	SCD > NC
ORBsup.R	ROL.R	−0.082 ± 0.036	−0.01 ± 0.027	0.004	SCD > NC
HIP.L	CUN.R	−0.076 ± 0.035	−0.012 ± 0.020	0.004	SCD > NC

*Note*: The NC and SCD columns represent the mean functional connectivity strength (FBN weights) between two brain regions. Positive values indicate positive functional connections, while negative values indicate negative functional connections. The “comparison (NC vs. SCD)” column indicates which group has a numerically greater mean FBN weight. Functional connectivity measurement and group comparison methods are consistent with previous studies (Y. Xu et al. 2019), where the strength of functional connectivity was used to differentiate between groups based on fMRI data. The statistical analysis was performed using Student's *t*‐test.

Abbreviations: AMYG, amygdala; CUN, cuneus; HIP, hippocampus; IFGoperc, inferior frontal gyrus, opercular part; IOG, inferior occipital gyrus; IPL, inferior parietal lobule; LING, lingual gyrus; MTG, middle temporal gyrus; OLF, olfactory cortex; ORBsup, superior orbital gyrus; PHG, parahippocampal gyrus; PoCG, postcentral gyrus; REC, rectus gyrus; ROL, Rolandic operculum; SPG, superior parietal gyrus; STG, superior temporal gyrus.

SRWT, similar to SR, highlights connections involving the middle temporal gyrus (MTG), medial orbitofrontal gyrus, and supramarginal gyrus. It also strengthens connections in the occipital gyrus, paracentral lobule (PCL), and fusiform gyrus (FFG). SRWT's integration of multiple frequency bands helps preserve critical cross‐frequency connectivity for SCD identification. Table 4 shows that the top 10 discriminative connections for SRWT are mainly in the temporal, parietal, and frontal lobes, linked to networks such as the DMN, FTC, and those related to memory and visual processing.

**TABLE 4:** Top 10 most discriminative connections for SRWT (*t*‐test based on *p* < 0.01).

Connection		NC	SCD	*p* value	Comparison (NC vs. SCD)
Region A	Region B				
ITG.R	ORBmid.L	−0.022 ± 0.030	0.058 ± 0.030	< 0.001	SCD > NC
CAU.R	ORBmid.R	−0.050 ± 0.035	0.037 ± 0.040	< 0.001	SCD > NC
AMYG.R	PCUN.R	−0.056 ± 0.077	0.062 ± 0.064	< 0.001	SCD > NC
PHG.R	CAL.R	−0.082 ± 0.072	0.025 ± 0.044	< 0.001	SCD > NC
CAU.R	CAL.R	0.022 ± 0.045	−0.087 ± 0.087	< 0.001	NC > SCD
SPG.L	PCUN.R	−0.077 ± 0.071	0.043 ± 0.088	< 0.001	SCD > NC
PCL.L	IPL.L	−0.057 ± 0.074	0.053 ± 0.062	< 0.001	SCD > NC
ORBsupmed.R	FFG.L	0.016 ± 0.066	−0.109 ± 0.107	0.002	NC > SCD
PUT.L	MOG.L	−0.028 ± 0.039	0.048 ± 0.027	0.002	SCD > NC
ORBsup.L	SFGmed.R	0.048 ± 0.047	−0.030 ± 0.030	0.002	NC > SCD

*Note*: The NC and SCD columns represent the mean functional connectivity strength (FBN weights) between two brain regions. Positive values indicate positive functional connections, while negative values indicate negative functional connections. The “comparison (NC vs. SCD)” column indicates which group has a numerically greater mean FBN weight.

Abbreviations: CAL, calcarine cortex; CAU, caudate; FFG, fusiform gyrus; ITG, inferior temporal gyrus; MOG, middle occipital gyrus; ORBmid, middle orbital gyrus; ORBsupmed, superior medial orbital gyrus; PCL, paracentral lobule; PCUN, precuneus; PUT, putamen; SFGmed, superior medial frontal gyrus.

Referring to our previous studies (X. Xu et al. 2021), we respectively identified the top 5% of brain regions with the largest weight as the hubs of group‐level brain networks based on PCWT and SRWT methods (Tables 5 and 6).

**TABLE 5:** Hubs of the NC and SCD groups based on the PCWT method.

	AAL number	Corresponding brain region	Anatomical classification	Subnetwork
NC	11	IFGoperc.L	Prefrontal	FTCN
	12	IFGoperc.R	Prefrontal	FTCN
	46	CUN.R	Occipital	VN
	61	IPL.L	Parietal	FTCN
	66	ANG.R	Parietal	DMN
	48	LING.R	Occipital	DMN
	44	CAL.R	Occipital	VN
	65	ANG.L	Parietal	DMN
	62	IPL.R	Parietal	FTCN
	68	PCUN.R	Parietal	DMN
SCD	66	ANG.R	Parietal	DMN
	51	MOG.L	Occipital	VN
	15	ORBinf.L	Prefrontal	DMN
	44	CAL.R	Occipital	VN
	47	LING.L	Occipital	DMN
	48	LING.R	Occipital	DMN
	43	CAL.L	Occipital	VN
	46	CUN.R	Occipital	VN
	65	ANG.L	Parietal	DMN
	13	IFGtriang.L	Prefrontal	FTCN

Abbreviations: AAL, automated anatomical labeling atlas; DMN, default mode network; FTCN, frontoparietal task control network; VN, visual network.

**TABLE 6:** Hubs of the NC and SCD groups based on the SRWT method.

	AAL number	Corresponding brain region	Anatomical classification	Subnetwork
NC	56	FFG.R	Temporal	DMN
	55	FFG.L	Temporal	DMN
	73	PUT.L	Subcortical	Subcortical
	29	INS.L	Limbic	SN
	89	ITG.L	Temporal	DMN
	74	PUT.R	Subcortical	Subcortical
	37	HIP.L	Limbic	Uncertain
	90	ITG.R	Temporal	DMN
	38	HIP.R	Limbic	Uncertain
	47	LING.L	Occipital	DMN
SCD	55	FFG.L	Temporal	DMN
	56	FFG.R	Temporal	DMN
	29	INS.L	Limbic	SN
	17	ROL.L	Frontal	CTCN
	73	PUT.L	Subcortical	Subcortical
	74	PUT.R	Subcortical	Subcortical
	89	ITG.L	Temporal	DMN
	90	ITG.R	Temporal	DMN
	37	HIP.L	Limbic	Uncertain
	30	INS.R	Limbic	SN

Abbreviations: AAL, automated anatomical labeling atlas; CTCN, cingulo‐opercular task control network; DMN, default mode network; SN, salience network.

### 3.4 Network‐Level Differences and Frequency‐Specific Patterns in SCD

To explore functional alterations in brain networks associated with SCD, we conducted a comparative analysis of FBNs generated by different methods. Despite methodological differences, both PCWT and SRWT consistently revealed clearer and more compact connectivity patterns between brain regions than their conventional counterparts. These refined representations may help better delineate altered functional organization in SCD.

To explore frequency‐dependent brain activity differences between SCD and NC groups, we applied an adaptive frequency selection approach (FSAWT). This method identified frequency bands that enhanced the discriminability of functional connectivity patterns. Both PCWT and SRWT consistently prioritized low‐frequency components (0.01–0.06 Hz), corresponding to core brain networks such as the default mode, sensorimotor, and visual systems. These findings suggest that frequency‐specific alterations may underlie early cognitive changes in SCD. Detailed visualizations and frequency decomposition results are provided in Supporting Information (2.2) and Figures S5 and S6.

## 4 Discussion

In this study, we identified distinct patterns of FBN alterations in individuals with SCD compared to cognitively NCs. Our results demonstrate that these network‐level differences—particularly within key regions such as the DMN, visual cortex, and limbic structures—can effectively distinguish individuals with early self‐perceived cognitive symptoms. Importantly, the classification models based on these functional signatures achieved high accuracy and sensitivity, underscoring their potential utility in identifying individuals at elevated risk for progression to AD. These findings highlight the value of functional connectivity biomarkers in the early detection and stratification of cognitive vulnerability.

Key biological markers identified include altered connectivity patterns, such as the reversal of PHG–SPG connections (from negative in NC to positive in SCD), which may reflect early DMN disintegration. This is clinically significant, as early detection could facilitate interventions to delay progression to AD, supported by recent findings of disrupted pDMN–PHG connectivity in SCD correlating with subjective memory complaints (Sharma et al. 2021). The study revealed network‐level alterations, particularly within the DMN, FTCN, and limbic systems. PCWT highlighted connections involving the PHG, SPG, and angular gyrus, suggesting disrupted integration between medial temporal and parietal regions. This aligns with the discussion of DMN's role in AD, noting early disruptions in PHG–parietal connectivity (Buckner et al. 2008). One study found that the SCD group showed stronger FC between the DMN seeds and the supramarginal gyrus compared to the MCI group. In addition, hyperconnectivity between the right lateral parietal cortex and the left supramarginal gyrus in the SCD group was significantly correlated with better performance on the Controlled Oral Word Association Test, which was distinct from the FC patterns observed in the MCI group (Lee et al. 2023). The observed PHG–SPG reversal may be associated with early DMN disintegration. One possible explanation is that such alterations could relate to Aβ deposition in DMN regions, as suggested by (Palmqvist et al. 2017), although this remains a speculative interpretation given the lack of neurochemical specificity in rs‐fMRI.

SRWT findings emphasized connections in temporal, parietal, and frontal lobes, such as FFG–orbitofrontal links, with low‐frequency (0.01–0.06 Hz) dominance suggesting slow oscillatory dysregulation. These patterns may be linked to changes in cross‐frequency coupling, which some studies have associated with tau‐related pathology (Vossel et al. 2013). However, such an interpretation should be considered hypothetical, as our rs‐fMRI data cannot directly address underlying molecular mechanisms. Hub analysis further revealed shifts, with SCD showing increased posterior DMN hubs, supported by findings of altered centrality frequency in SCD, particularly in posterior DMN regions (Xie et al. 2019).

Research has shown that in AD, hub regions, particularly within the DMN, are vulnerable to pathology due to their high metabolic activity. Previous studies identified cortical hubs and their relation to AD pathology, and also demonstrated disrupted modular dynamics in AD, suggesting hub vulnerability (Buckner et al. 2009; de Haan et al. 2012). In preclinical stages like SCD, alterations in hub regions may be more subtle, potentially involving compensatory increases in connectivity or shifts to different networks (Viviano and Damoiseaux 2020). Our findings of altered hub distributions in SCD are consistent with reports of reconfigured brain network dynamics in SCD, such as increased occupancy in less activated states of certain networks (Chen et al. 2021).

The introduction of FSAWT, with dynamic frequency selection, addresses the “subtlety” detection bottleneck in SCD. By capturing connections like PHG–SPG and FFG–orbitofrontal, missed by fixed‐band methods, PCWT and SRWT enhance diagnostic efficacy. This is a methodological leap over traditional approaches, with previous work highlighting fixed‐band limitations (Zuo et al. 2010). PCWT's parameter robustness supports clinical deployment, while SRWT's high accuracy suits high‐risk screening, aligning with an individualized connectomics framework (Seidlitz et al. 2018).

Conventional methods assume stationarity and rely on fixed frequency bands, overlooking the dynamic and frequency‐dependent nature of brain connectivity (Chang and Glover 2010; Yaesoubi et al. 2015). Although wavelet‐based and dynamic connectivity approaches improved sensitivity, they often require fixed bands or high computational cost (Achard et al. 2006; Bassett et al. 2011). Our FSAWT method overcomes these issues by adaptively selecting “golden frequencies,” achieving higher accuracy and revealing biologically plausible patterns, such as PHG–SPG reversal and low‐frequency dominated FFG–orbitofrontal hyperconnectivity, consistent with AD‐related disruptions.

These methods hold promise for early, noninvasive SCD screening, potentially integrating with PET biomarkers for AD risk stratification. Future research should focus on longitudinal studies to assess predictive value for AD conversion, as suggested by the cross‐sectional nature of current findings. Validation in community cohorts is essential to ensure SRWT parameter stability and generalizability.

Several limitations warrant consideration. First, demographic confounding, with SCD group being younger and having more females, may bias results, necessitating age‐matched analyses or covariate adjustments. Second, the lack of longitudinal data limits causal inference regarding connectivity changes and AD progression. Third, SRWT's parameter sensitivity requires validation in larger samples to ensure clinical applicability.

## 5 Conclusion

The study concludes that FSAWT offers a robust framework for early SCD detection by integrating frequency‐specific and cross‐frequency dynamics, advancing precision diagnostics and personalized interventions for neurodegenerative disorders.

## Author Contributions

Conceptualization: Weikai Li and Xiaowen Xu. Data curation: Mingyu Tan, Peiying Chen, Zhongfeng Xie, Mengling Tao, Yongsheng Xiang, and Yingying Liu. Formal analysis: Xiereniguli Anayiti, Yupan Ding, and Xiaowen Xu. Funding acquisition: Peijun Wang. Methodology: Xiereniguli Anayiti, Yupan Ding, Weikai Li, Mingyu Tan, Peiying Chen, Zhongfeng Xie, Mengling Tao, Yongsheng Xiang, Yingying Liu, and Xiaowen Xu. Project administration: Peijun Wang. Resources: Peijun Wang. Software: Yupan Ding, Weikai Li, and Yongsheng Xiang. Supervision: Xiaowen Xu and Peijun Wang. Visualization: Weikai Li. Writing – original draft: Xiereniguli Anayiti. Writing – review and editing: Xiaowen Xu.

## Consent

The study was approved by the Ethics Committee of Tongji Hospital affiliated with Tongji University (ChiCTR2000030614). Written informed consent was obtained from all participants. All BEC participants provided written informed consent in compliance with the Declaration of Helsinki.

## Conflicts of Interest

The authors declare no conflicts of interest.

## Supporting information




**Supplementary Material**: brb371039‐sup‐0001‐SuppMatt.docx

## Data Availability

All data generated or analyzed during this study are included in this published article.
