# Supplementary material for: Adaptive Frequency‐Optimized Wavelet Networks for Early Detection of Subjective Cognitive Decline via Resting‐State fMRI
Source: Brain Behav. 2025 Dec 7;15(12):e71039. doi: 10.1002/brb3.71039 (PMC12683069; doi:10.1002/brb3.71039)
Supplement: Supplementary file 1 — Supplementary Material: brb371039‐sup‐0001‐SuppMatt.docx [file BRB3-15-e71039-s001.docx]

**1.Materials and Method**

**1.1** **Methods in brain network analysis**

Most research in this field has focused on correlation-based methods, like Pearson Correlation (PC), which estimate associations between brain regions. However, these methods often produce fully connected networks, introducing redundant or irrelevant links. To address this, thresholding or regularization is applied to create sparse functional brain networks (FBNs). Sparse and low-rank constraints are also used to identify modular structures in FBNs. Some researchers use spatial wavelet transforms to decompose resting-state fMRI data into frequency components(Luo et al., 2020). This technique leverages spatial correlations for efficient encoding and captures complex, non-stationary patterns, improving reliability.

Pearson correlation is a fully connected network structure that captures interactions between brain regions by calculating correlations between nodes. During the preprocessing of rs-fMRI sequences, the brain was partitioned into ROIs, with each ROI corresponding to a time point. We represented the data as a matrix, with columns indicating the time series of ROIs and rows corresponding to time points. Figure S1(A)① illustrates the weight matrix of the FBN. By centering and normalizing this matrix, PC can be expressed in matrix form. PC is estimated through a least squares method using the L2 norm, with balance controlled as shown in Equation ② of Figure S1(A). To mitigate noise caused by densely connected networks in the BOLD signal, thresholding is applied to the PC-derived correlation matrix, resulting in a filtered FCN, as shown in Equation ④ of Figure S1(A).

Partial correlation quantifies the direct relationship between two variables by removing the effects of others. Unlike the principal component approach, which includes confounding influences, partial correlation isolates direct associations, offering a clearer view of variable relationships. Typically, the partial correlation between $x_{i}$ and $x_{j}$ can be denoted by the inverse of the sample covariance matrix $\Sigma$, which is also known as the precision matrix $\Pi$ in Figure S1(B)① ②,where $w_{ij}$ denotes the partial correlation coefficient between $x_{i}$ and $x_{j}$, and $\Pi=\left[ \pi_{ij} \right]$. Due to the singularity of the covariance matrix and the small sample size issue in FBN estimation (where the number of fMRI time points is much smaller than the number of eigenvectors), the task of estimating partial correlation coefficients is reformulated as a regression problem, utilizing sparse representation and LASSO ：Figure S1(B) ③.

The Continuous Wavelet Transform (CWT) is a technique for analyzing signal frequency across multiple scales by transforming it from the time domain to the time-scale domain using wavelet functions. In rs-fMRI, CWT provides detailed information about both temporal and frequency variations, capturing subtle changes in the time-series signal. The formula for CWT is presented in Figure S1(C)①.


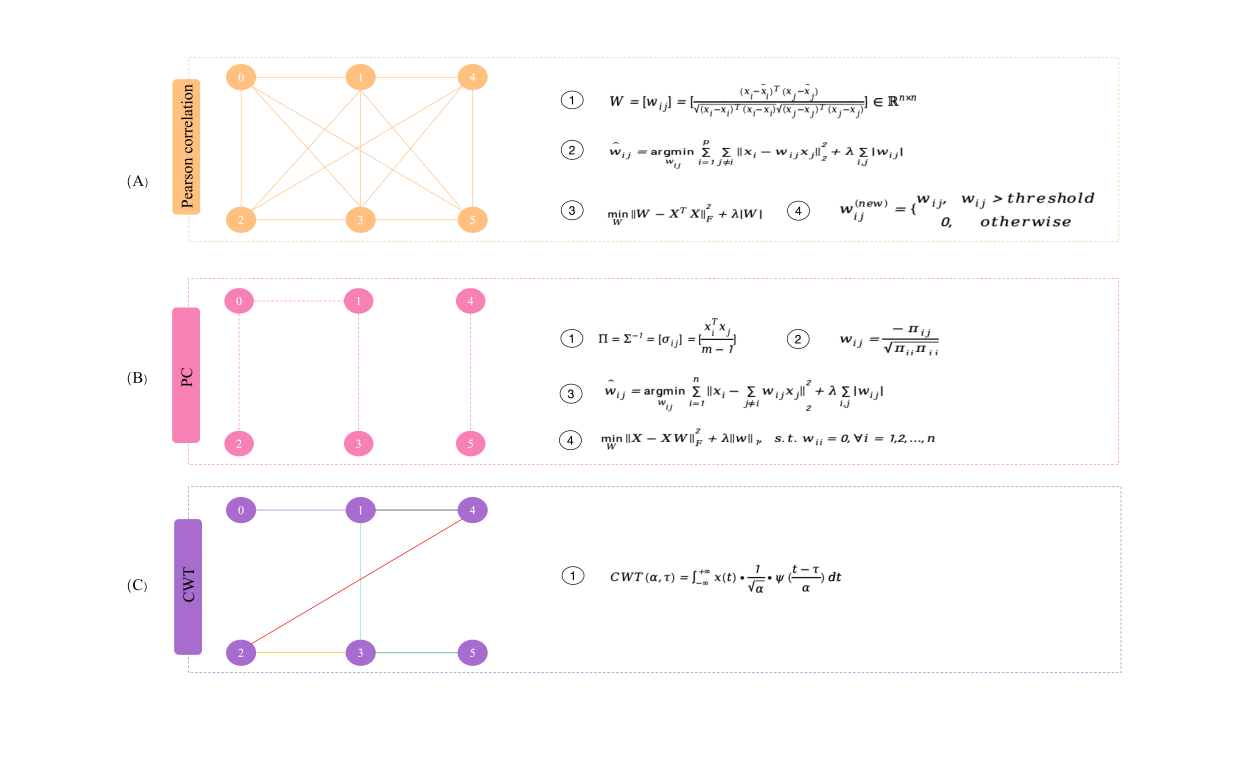


**Figure S1 Comparison of graph-based methods for modeling pairwise relationships and their corresponding equations: Pearson correlation (A), probabilistic covariance (B), and continuous wavelet transform (C).**

**1.2 Functional network optimization via frequency self-adaptive wavelet transform**

Fixed frequency bands can distort FBNs due to varying regional responses, and sinusoidal functions fail to capture transient rs-fMRI phenomena. This study proposes a dynamic approach to optimize frequency responses for more accurate brain connectivity mapping.

**(1) Enhancing FBN Construction.** To introduce the proposed wavelet, transform-based time-series frequency self-adaptive model, we begin with an example using an adjacency matrix. Rs-fMRI data from subjects with normal cognitive function (NC) and SCD were divided into 90 ROIs (AAL template), each with 80 time points. Traditional FBN construction methods relying on preprocessed fMRI data or fixed frequency bands often fail to capture complex frequency dynamics, leading to spurious connections. As shown in the left panel of Figure S2, only a few regions exhibit clear "blocky" topological structures, while most areas display mixed patterns, highlighting significant noise in adjacency matrices derived from PC. A noisy adjacency matrix complicates FBN interpretation. To address this, we decompose ROI signals into frequency components and construct correlation matrices across scales. By optimizing region groupings, we generate clearer matrices with distinct partitions. As shown in Figure S2 (right), the method enhances the identification of strongly correlated regions while reducing noise. To evaluate the restructured matrix, we use the Brain Connectivity Toolbox (BCT) and modularity. The PCWT-generated matrix shows a 9.19% average improvement in modularity, with a 14.14% increase at a 10% threshold.


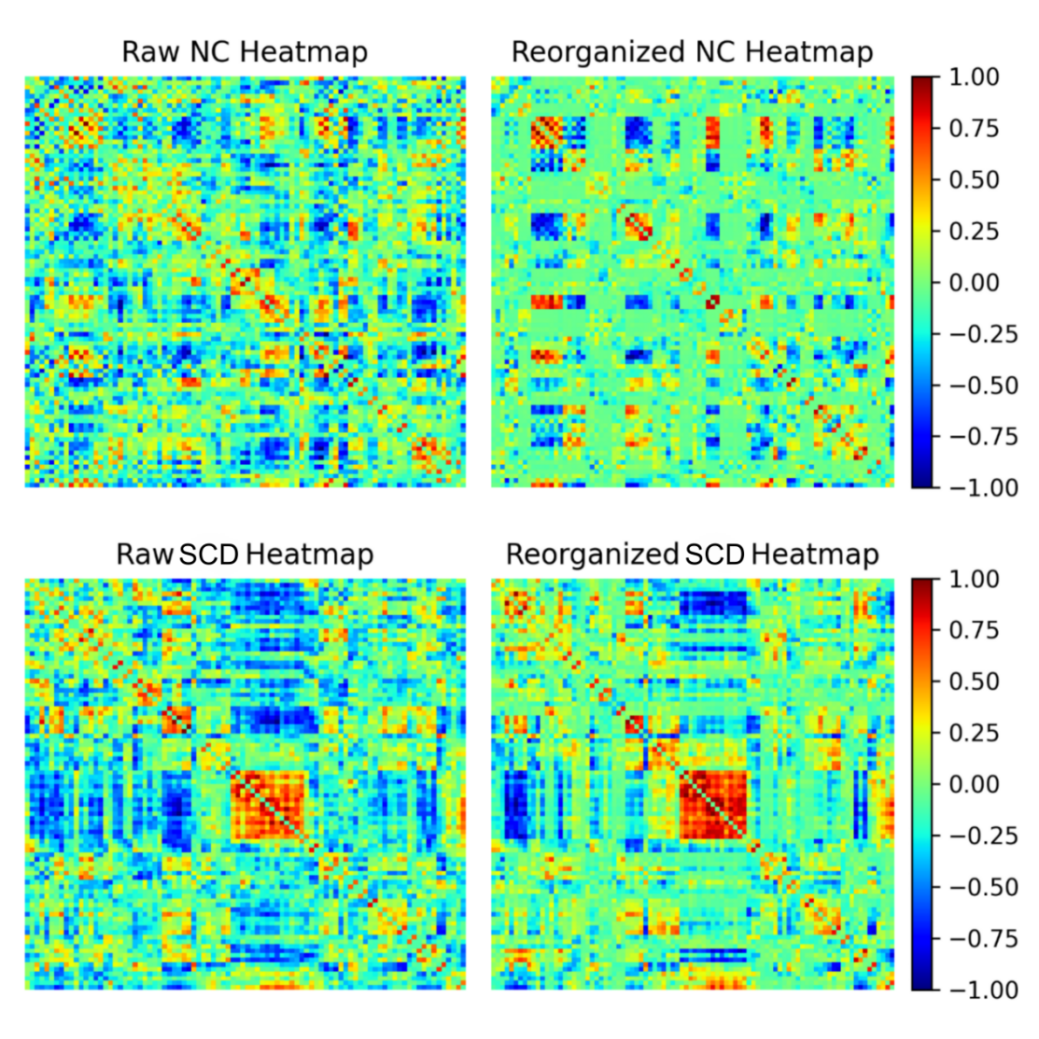


**Figure S2. Heatmap of randomly selected NC and SCD (Raw NC and SCD on the left, and NC and SCD processed by the PCWT method on the right)**

**(2) Model.** Assume the $col$-th ROI of the $i$-th subject is denoted as $X^{\left( i,col \right)}=x_{col}^{\left( i \right)}\in\mathbb{R}^{m\times1},i=1,2,\ldots; col=1,2,\ldots,n$. Using equation in Figure S1(C)①，we dissect the components of this column of ROI using wavelet transform, expressed as ${CWT}^{\left( i,col \right)}\left( \alpha_{k},\tau_{l} \right)$. For simplicity, we rewrite this as：

| $\left[ {CWT}^{\left( i,col \right)}\left( \alpha_{k},\tau_{l} \right) \right]^{T}=\left[ c_{1}^{\left( i,col \right)},c_{2}^{\left( i,col \right)},\ldots,c_{a}^{\left( i,col \right)} \right]\in\mathbb{R}^{m\times a}$ |
| --- |
| $c_{k}^{\left( i,col \right)}=\left( {CWT}^{\left( i,col \right)}\left( \alpha_{k},\tau_{1} \right),{CWT}^{\left( i,col \right)}\left( \alpha_{k},\tau_{2} \right),\ldots,{CWT}^{\left( i,col \right)}\left( \alpha_{k},\tau_{m} \right) \right)^{T}\in\mathbb{R}^{m\times1}$ |

where, $k=1,2,\ldots,a$, $a$ represents the wavelet scales defined in the previous section, and $m$ indicates the number of time points in the rs-fMRI data acquisition. By applying the wavelet scale associated with the minimum frequency to decompose each ROI of the first subject, a new matrix $\tilde{X}^{\left( 1 \right)}=\left( c_{1}^{\left( 1,1 \right)},c_{1}^{\left( 1,2 \right)},\ldots,c_{1}^{\left( 1,n \right)}\in\mathbb{R}^{m\times n} \right)$ is formed, with $n$ denoting the total number of ROIs.

To reduce computational complexity, we introduce a scaling factor $\beta\mathbb{\in Z}$ to divide ROIs into groups of size $\frac{n}{\beta}$. By setting $a=20$ and $\beta=18$, the 90 ROIs can be divided into 18 groups, each containing 5 ROIs. As a result, this partitioning decreases the number of combinations from ${20}^{90}$ to $18\times{20}^{5}$, effectively lowering the time complexity from $O\left( N^{90} \right)$ to $O\left( N^{5} \right)$. By incorporating the $\tilde{X}$ derived from our approach into equation in Figure S1(A)③, the PC method based on wavelet transform augmentation of the original data is formulated as follows:

$$\min_{W} \left\| W-\tilde{X}^{T}\tilde{X} \right\|_{F}^{2}+\lambda\left| W \right|$$

**(3) Algorithm.**

Continuous wavelets transform (CWT) as described in Equation（Figure S1(C)①）is primarily implemented using MATLAB's Wavelet Toolbox. Before that, we initialize matrices $\tilde{X}$ and $\tilde{W}$ as empty matrices. These matrices are utilized to generate the PC matrix of the preprocessed rs-fMRI sequence for comparison and subsequent storage in the following iterations.

**Step 1:** Using the scaling factor $\beta$, the input matrix $X$ is divided into multiple groups. The time series signals in each group are analyzed through wavelet decomposition. Equation in Figure S1(C)① transforms time-domain signals in each column into frequency-domain signals across multiple bands, capturing distinct features of each frequency component.

**Step 2:** Given our focus on· $\max_{\tilde{W}} \left| w^{ij} \right|$, we construct empty matrices $\tilde{X}$ and $\tilde{W}$. Subsequently, the decomposed frequency-domain signals and the original time series from each column are individually populated into the empty columns. The Pearson correlation coefficients $\left[ w^{ij} \right]$ for each combination are then computed.

**Step 3:** This process is repeated for each group, retaining the combination with the highest absolute values to update the matrix$\tilde{X}$. This procedure is summarized in Algorithm 1.

The final result $\tilde{X}$ obtained from the frequency self-adaptive wavelet decomposition will be used as input for PC and SR, followed by gradient descent optimization. Particularly, we introduce the functional forms of the data-fitting terms, defined in Equation(Figure S1(A)③ and (B)④)as: $f^{\left( PC \right)}\left( \tilde{X},W \right)=\left\| W-\tilde{X}^{T}\tilde{X} \right\|_{F}^{2}$ and $f^{\left( SR \right)}\left( \tilde{X},W \right)=\left\| \tilde{X}-\tilde{X}W \right\|_{F}^{2}$.The gradients of these functions are$\nabla_{W}f^{\left( PC \right)}\left( \tilde{X},W \right)=2\left( W-\tilde{X}^{T}\tilde{X} \right)$and $\nabla_{W}f^{\left( SR \right)}\left( \tilde{X},W \right)=2\left( \tilde{X}^{T}\tilde{X}W-\tilde{X}^{T}\tilde{X} \right)$.Based on the gradient descent criterion, $F$can be updated using the following equation.

| $W_{k}=W_{k-1}-\alpha_{k}\nabla_{W}f\left( \tilde{X},W_{k-1} \right)$ |
| --- |

In this context, $\nabla_{W}f$is determined by either PC or SR, while $\alpha_{k}$ denotes the step size used in the gradient descent process, with an initial value set to 0.001. More comprehensive details on the adaptive updating procedure can be found in the SLEP toolbox (<http://www.yelab.net/software/SLEP>).

To mitigate issues such as the convergence of Equation (Figure S1(A)③) to a local optimal or the vanishing of gradients, we adopt the proximal method. The proximal operator is defined by the following equation:

| $prox\left( W \right)=\left[ sgn\left( w_{ij} \right)\times max\left( \left\vert w_{ij} \right\vert-\lambda,0 \right) \right]_{n\times n}$ |
| --- |

Here, $sgn\left( \cdot\right)$ denotes the step function, which is used to determine the sign of $\tilde{w}_{ij}$. The algorithm for estimating the FBN based on PC is presented in Algorithm 2.

**1.3 Experimental Setting**

Following preprocessing, the MRI dataset is condensed to 80 time points. Additionally, we use the AAL template to partition the BOLD signals of each subject into 90 ROIs. To assess the performance of the proposed method, we benchmark it against two baseline techniques, PC and SR. The data is processed using FSAWT and then compared with PCWT and SRWT.

To identify the optimal scale for wavelet decomposition, the parameter $a$ is selected using Equation (Figure S1(C)①), which divides the BOLD time-domain signal into 20 distinct frequency bands. Given that the dataset has a repetition time (TR) of 3000 ms and a sampling frequency of $F_{s}=\frac{1}{3}\approx0.333$Hz. Consequently, the relationship for the $i$-th decomposition signal at the first scale is expressed as:

| $F_{i}=\frac{F_{c}\times F_{s}}{i}, i=1,2,\ldots,20$ |
| --- |

In this context, $F_{c}$ represents the central frequency of the wavelet. Specifically, the central frequency of the sym4 wavelet is 0.714 Hz, while the central frequency of the db6 wavelet is 0.7273 Hz.

More specifically, smaller scales correspond to higher frequencies, indicating more pronounced wavelet function characteristics and finer decomposition of the signal, which can capture rapid variations in the original signal over shorter time intervals. In contrast, larger scales correspond to lower frequencies, representing smoother wavelet function characteristics and a coarser decomposition of the signal, which captures the overall trend of the data.

For resting-state brain functional data, low-frequency oscillations within the 0.01-0.08Hz range contain crucial information that reflects spontaneous neural activity during rest(Bailes et al., 2023). To preserve the data's topological structure and reduce noise, we exclude the first four scales, as they mainly capture high-frequency components that often include noise(Halidou et al., 2023). After selecting the relevant wavelet decomposition scales and frequency bands, we use Algorithm 1 to find the optimal combination. This combination is then applied in PC and SR, resulting in the PCWT and SRWT methods. For PC and PCWT, we set the threshold values as$\left[ 0,0.1,0.2,\ldots,0.9,0.95,0.99 \right]$, use the sym4 wavelet function, and set the scaling factor to $\beta=18$. For SR and SRWT, the parameter $\lambda$ is chosen from the range $\left[ 2^{-5},2^{-4},\ldots,2^{0},\ldots,2^{4},2^{5} \right]$, with the db6 wavelet function and $\beta=30$.

After obtaining the FBN using PC, PCWT, SR, and SRWT for all subjects, we select relevant features to distinguish NC and SCD. To reduce feature dimensionality, we extract the upper triangular elements of the symmetric FBN and perform a t-test at p-values of 0.05, 0.01, and 0.005, choosing the one with the best classification performance. For classification, we use a simple linear classifier (linear kernel with C=1) to focus on the impact of data modification. More complex classifiers may mask improvements from data changes, and medical imaging often involves small sample sizes, increasing the risk of overfitting with more complex models.

To reduce overfitting, we use a combination of leave-one-out (LOO) and nested cross-validation for training and hyperparameter optimization. In this approach, the data is split into internal and external layers. The internal layer performs LOOCV to find optimal hyperparameters, while the external layer also uses LOOCV to evaluate the test set with the internal hyperparameters. Finally, we assess classification performance using metrics like accuracy, sensitivity, specificity, and the Area Under the Curve (AUC) from the ROC curve for comparison.

Additionally, the average value of the individual brain network was acquired to generate the group-average network. We identified the hub nodes by ranking the nodal degree. The rank 5% of brain regions were defined as the hubs of the brain network(Xu et al., 2024; Zhao et al., 2020).

**2.Results**

**2.1 Diagnostic performance of SCD classification**

The classification accuracy of PC and SR methods is sensitive to hyperparameter choices. Parameter experiments revealed that SRWT is more sensitive to parameter variations than PCWT. Specifically, the range of p-values was defined as [0.005,0.01,0.02,…,0.09,0.1], and the performance of the two methods under different hyperparameters was examined (Figure S3 and Figure S4). This heightened sensitivity can be attributed to the intrinsic nature of LASSO’s penalty term, which enforces sparsity by constraining the sum of the absolute values of the coefficients. Importantly, PCWT achieved its highest accuracy of 75.33% with a threshold of 50 and a p-value of 0.03, whereas SRWT reached its peak accuracy of 84.11% with λ=2^0 and a p-value of 0.01.


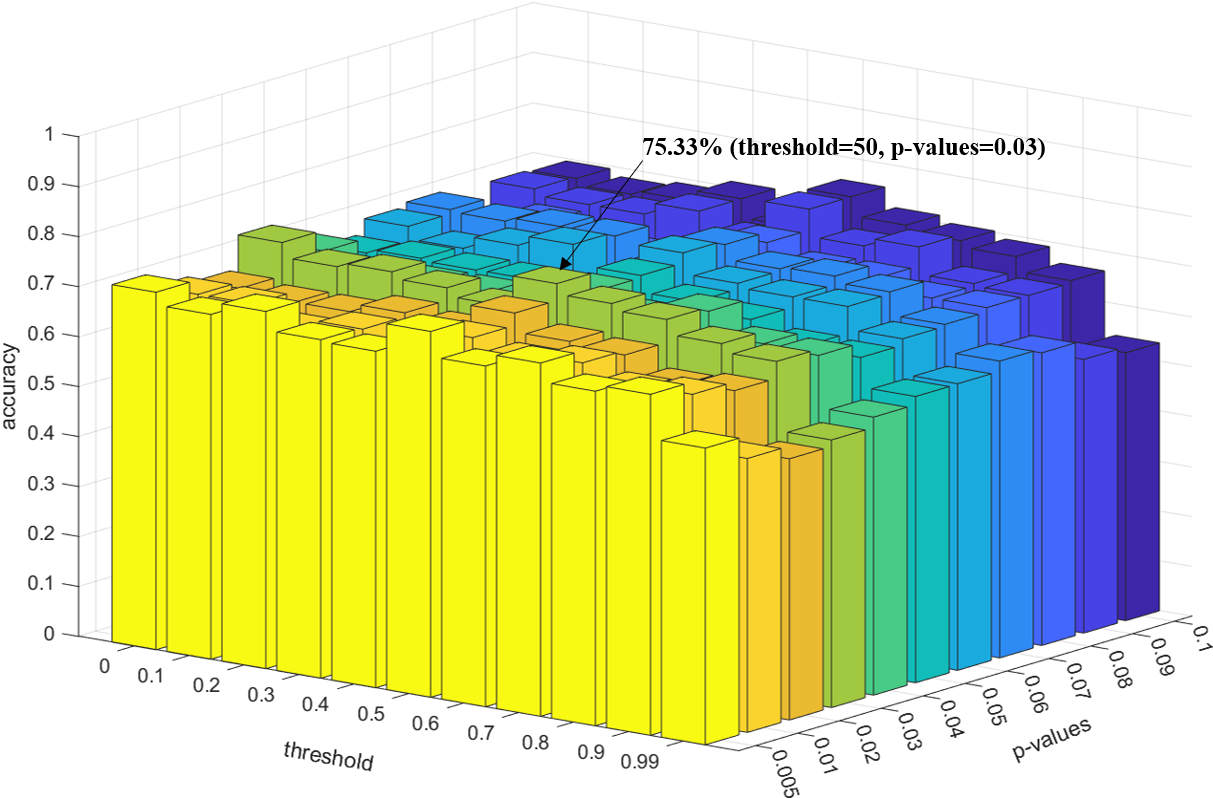


**Figure S3. Classification results of PCWT for different thresholds and p-values.**


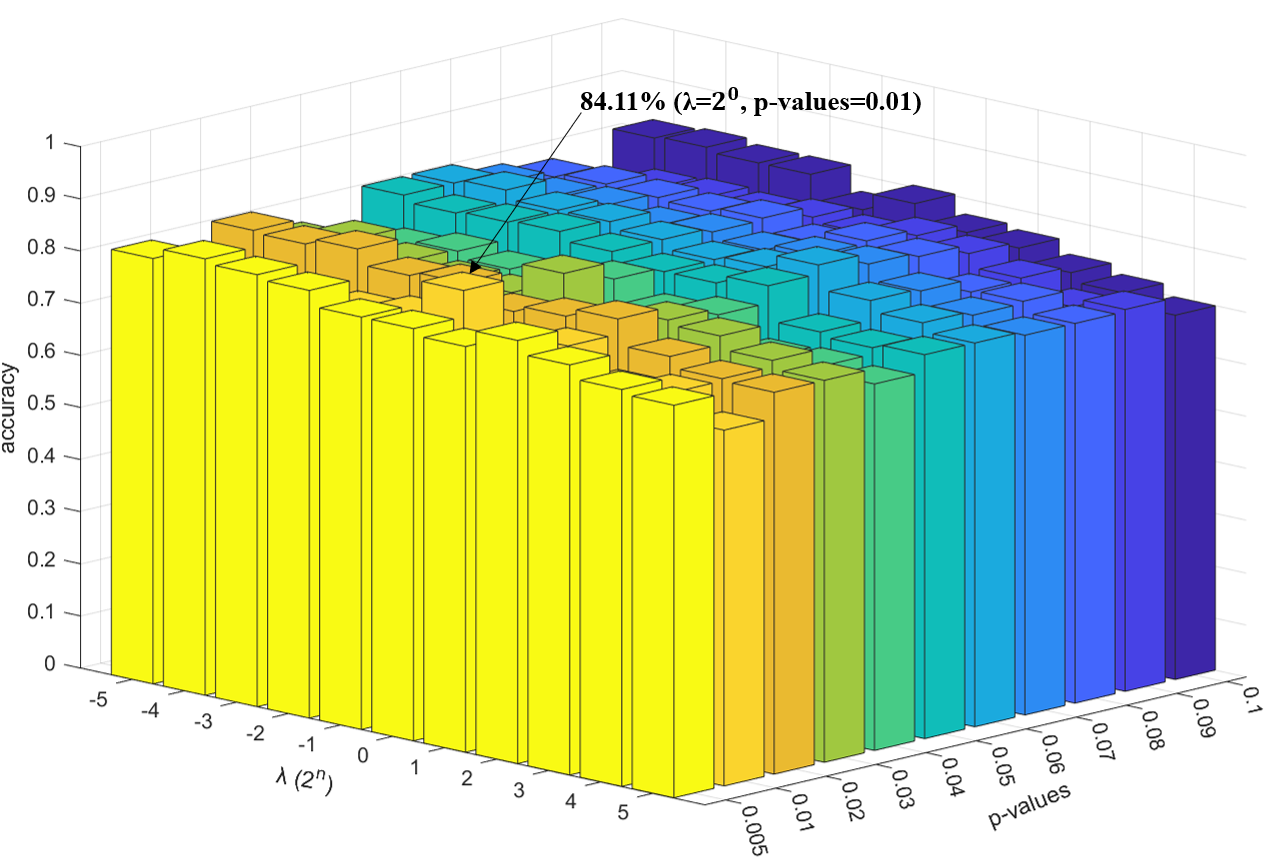


**Figure S4. Classification results of SRWT for different** $\lambda$ **and p-values.**

**2.2 Network-level Differences and Frequency-Specific Patterns in SCD**

We analyze the differences in FBN adjacency matrices created by PC, PCWT, SR, and SRWT methods. The FBN matrix from the PC method is derived by applying a threshold to the PC coefficients between features. Figure S5 shows visualizations of FBN weight networks created using the four methods. For the PC and PCWT approaches, a 20% quantile threshold is applied, while the value $\lambda=2^{-2}$ is used for the SR and SRWT methods. However, PCWT reduces color-mixed regions. PC's all-connectivity feature highlights both strongly and weakly correlated areas, causing color mixing in certain parts of the heat map, which could affect classification. In contrast, after PCWT processing, strongly correlated regions appear more compact and vividly colored, while weak correlations are further reduced, leading to a lighter overall appearance in some areas. The adjacency matrix from SR differs from those of PC and PCWT, as it uses sparse representation-based partial correlation instead of Pearson full correlation. Similarly, SRWT's matrix is similar to SR's but with more pronounced connections, likely due to SRWT emphasizing deeper cross-frequency relationships, resulting in increased inter-regional connectivity.


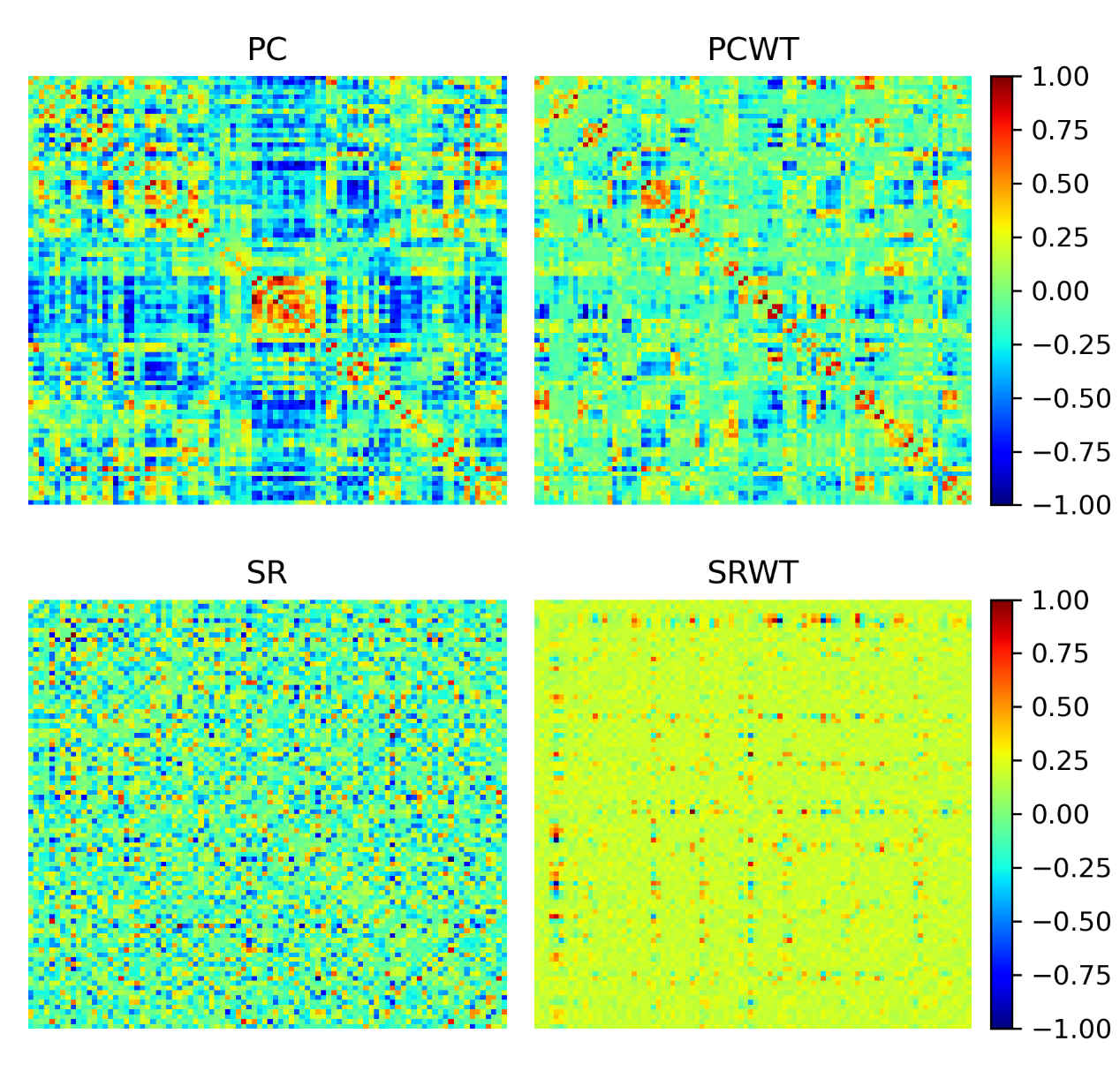


**Figure S5. FBN weighting network for PC, PCWT, SR and SRWT methods for the same subjects, where subjects are randomly selected (all weights are normalized between -1 and 1 to facilitate comparison between methods).**

FSAWT adapts to select the frequency combination with the highest Pearson correlation coefficient, mapping brain responses across frequencies. When this combination is more informative than the original signal, its decomposed components replace the initial signal. The process aims to identify "golden frequencies" that reveal distinct connectivity patterns between SCD and NC. This study seeks to pinpoint key frequency bands that differentiate brain responses in SCD and NC, providing insights into the underlying neural mechanisms. Figure S6 presents a statistical analysis of the frequencies replaced by PCWT and SRWT, highlighting the differences between SCD and NC. For SCD subjects, PCWT highlights the fifth frequency band at 0.0727Hz, with original signals mainly decomposed within a specific frequency interval. SRWT emphasizes the twentieth frequency band at 0.0179 Hz, with signals decomposed across all selected frequency bands. For NC subjects, PCWT accentuates the fifth frequency band at 0.0727 Hz, and SRWT again focuses on the twentieth frequency band, with decomposition similar to that of SCD. Both methods prioritize frequencies between 0.01 Hz and 0.06 Hz, corresponding to networks such as the sensorimotor, default mode, and visual cortex.


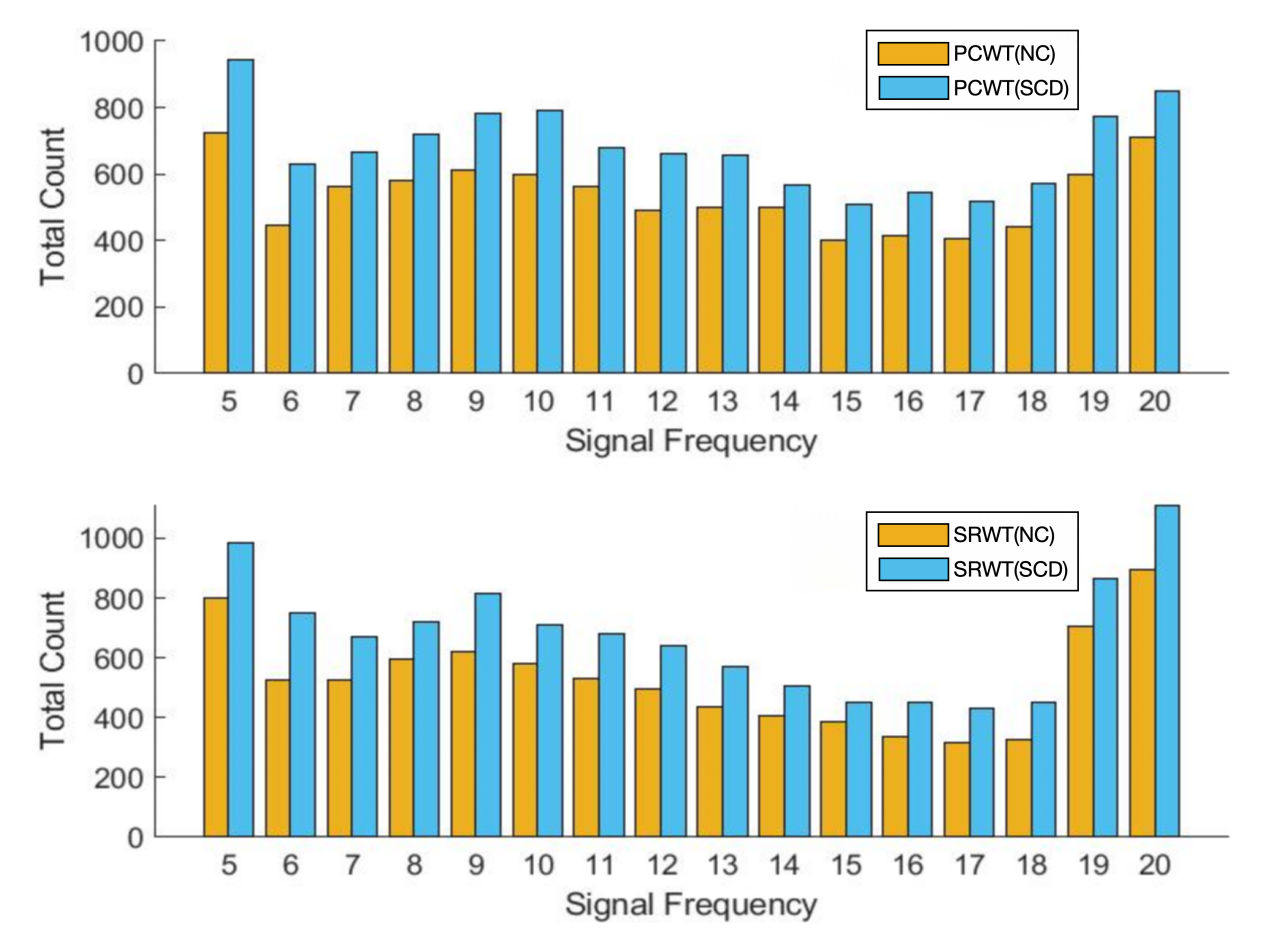


**Figure S6. The number of frequencies selected between NC and SCD for PCWT and SRWT, where 5 to 20 for PCWT corresponds to the different frequencies decomposed by the db6 wavelet in the previous section, and 5 to 20 for SRWT corresponds to the different frequencies decomposed by the sym4 wavelet in the previous section.**

Bailes, S. M., Gomez, D. E., Setzer, B., & Lewis, L. D. (2023). Resting-state fMRI signals contain spectral signatures of local hemodynamic response timing. *eLife*, *12*, e86453. https://doi.org/10.7554/eLife.86453

Halidou, A., Mohamadou, Y., Ari, A. A. A., & Zacko, E. J. G. (2023). Review of wavelet denoising algorithms. *Multimedia Tools and Applications*, *82*(27), 41539–41569. https://doi.org/10.1007/s11042-023-15127-0

Luo, F.-F., Wang, J.-B., Yuan, L.-X., Zhou, Z.-W., Xu, H., Ma, S.-H., Zang, Y.-F., & Zhang, M. (2020). Higher Sensitivity and Reproducibility of Wavelet-Based Amplitude of Resting-State fMRI. *Frontiers in Neuroscience*, *14*, 224. https://doi.org/10.3389/fnins.2020.00224

Xu, X., Chen, P., Li, W., Xiang, Y., Xie, Z., Yu, Q., Tang, Y., & Wang, P. (2024). Topological properties analysis and identification of mild cognitive impairment based on individual morphological brain network connectome. *Cerebral Cortex (New York, N.Y.: 1991)*, *34*(1), bhad450. https://doi.org/10.1093/cercor/bhad450

Zhao, W., Guo, S., Linli, Z., Yang, A. C., Lin, C.-P., & Tsai, S.-J. (2020). Functional, Anatomical, and Morphological Networks Highlight the Role of Basal Ganglia-Thalamus-Cortex Circuits in Schizophrenia. *Schizophrenia Bulletin*, *46*(2), 422–431. https://doi.org/10.1093/schbul/sbz062
